# Validation of reference genes for quantitative RT-PCR studies in porcine oocytes and preimplantation embryos

**DOI:** 10.1186/1471-213X-7-58

**Published:** 2007-05-31

**Authors:** Ewart W Kuijk, Leonie du Puy, Helena TA van Tol, Henk P Haagsman, Ben Colenbrander, Bernard AJ Roelen

**Affiliations:** 1 Department of Farm Animal Health, Faculty of Veterinary Medicine, Utrecht University, Yalelaan 104, 3584 CM Utrecht, The Netherlands; 2 Department of Infectious Diseases and Immunology, Faculty of Veterinary Medicine, Utrecht University, Yalelaan 1, 3584 CL Utrecht, The Netherlands

## Abstract

**Background:**

In the developing embryo, total RNA abundance fluctuates caused by functional RNA degradation and zygotic genome activation. These variations in the transcriptome in early development complicate the choice of good reference genes for gene expression studies by quantitative real time polymerase chain reaction.

**Results:**

In order to identify stably expressed genes for normalisation of quantitative data, within early stages of development, transcription levels were examined of 7 frequently used reference genes (*B2M, BACT, GAPDH, H2A, PGK1, SI8*, and *UBC*) at different stages of early porcine embryonic development (germinal vesicle, metaphase-2, 2-cell, 4-cell, early blastocyst, expanded blastocyst). Analysis of transcription profiling by geNorm software revealed that *GAPDH, PGK1, S18*, and *UBC *showed high stability in early porcine embryonic development, while transcription levels of *B2M, BACT*, and *H2A *were highly regulated.

**Conclusion:**

Good reference genes that reflect total RNA content were identified in early embryonic development from oocyte to blastocyst. A selection of either *GAPDH *or *PGK1*, together with ribosomal protein *S18 *(*S18*), and *UBC *is proposed as reference genes, but the use of *B2M, BACT*, or *H2A *is discouraged.

## Background

Preimplantation development is a highly dynamic process including phenomena such as oogenesis, oocyte maturation, fertilization and lineage segregation. Not surprisingly, early embryonic development is characterized by dramatic changes in transcription. To start with, at the diplotene stage of an oocyte's first meiotic prophase, numerous genes are transcribed and translated, resulting in storage of mRNA and proteins that support early embryonic development. After completion of maturation, oocytes arrest at the metaphase of the second meiotic division, where transcription is halted and translation of mRNA is reduced [1]. Transcription is restored after fertilization, which can be detected by incorporation of labelled nucleotides in mRNA of embryos [2,3]. In mammals, the start of zygotic genome activation varies between 1- and 8-cell stage embryos, dependent on the species [4].

The main part of our knowledge on the transition from maternal to zygotic transcipts has been derived from studies in mouse, but also from studies in rabbits, cattle and pigs. In the mouse, early embryonic development shows wave-like gene activation patterns and two major transitions in gene expression can be recognized. The first major wave of gene activation peaks at the 2- to 4-cell stage. This corresponds to the well-known zygotic genome activation, by which embryonic transcripts replace maternally inherited transcripts. The second major wave of gene activation, the so-called mid-preimplantation genome activation, peaks at the 8-cell stage and precedes the obvious morphological changes in subsequent stages: compaction and blastocyst formation. These major waves of activation are followed by two minor waves, one at the morula stage and another at the blastocyst stage [5]. In addition to these bursts in transcription, maternal mRNA is actively degraded during early embryonic development, restricting the time window in which these transcripts can function. In the mouse, the amount of total RNA at the 2-cell stage is reduced to ~10% compared to that of an unfertilised oocyte [5-7]. These temporal changes in transcriptome are considered to fulfil the need of the embryo for particular classes of proteins at the appropriate phases in development. Minor genome activations, prior to the major zygotic genome activation, have been observed during the first cleavage stages of rabbit, human, and bovine embryos, which suggests a conserved mechanism of sequential acquisition of transcriptional control in mammals [8-13].

Dynamic changes in the transcriptome take place in the developing porcine embryo as well. In this species, loss of maternal transcripts in zygotes is demonstrated by decreasing levels of *CYCLIN B1 *and *CDC25C *in early embryonic development [14,15]. At the 4- cell stage of porcine embryos the first synthesis of extra-nucleolar RNA was observed by using uridine-3H labelling techniques, which is an indication that from the third cell cycle onwards the porcine embryonic genome is reactivated. [16]. Activation of the porcine embryonic genome at this stage of development is also confirmed by fluorescent in situ hybridisation of rRNA transcripts at the 4-cell stage but not in earlier embryonic stages [17]. *CDC25C *transcription is also observed in 4-cell stage pig embryos [14], another indication that the porcine embryonic genome is active in these embryos. In other words, early porcine embryonic development is characterized by significant changes in the transcriptome.

Studying alterations in gene expression is essential to understand the processes that are important for preimplantation development. Presently, the method of choice to quantify mRNA levels is quantitative Reverse Transcription Polymerase Chain Reaction (qRT-PCR), particularly when small amounts of mRNA are present [18]. In gene expression studies, there are several sources of variation, such as the amount of starting material, enzymatic efficiencies, and differences between tissues or cells in overall transcriptional activity. In an ideal situation, transcript numbers are standardized to the number of cells [19]. However, during preimplantation development, cell numbers and cell sizes are constantly changing. Alternatively, gene expression is normalized by the amount of starting material, but RNA yield from preimplantation embryos is too low for reliable quantification and variation in its reverse transcription cannot be excluded. Also for proper use of external controls, quantification of starting material is indispensable [20], and therefore not the preferred method when using small amounts of material. An elegant way to control for all variables, including the amount of input material, is normalization against internal control genes, on the condition that these internal control genes are stably expressed [19]. Traditionally, mRNA levels in Northern blots and RNAse protection assays have been quantified by normalisation to a reference gene that shows constant expression levels between samples with similar RNA content. In the present study, a similar line is followed; a stably expressed gene is defined as a gene of which its quantity is an indication of the total amount of RNA. In other words, a good reference gene for early embryonic development reflects the fluctuating transcriptomes by its expression. Commonly used reference genes are beta-actin (*BACT*), glyceraldehyde-3-phosphate dehydrogenase (*GAPDH*), and 18S ribosomal RNA (*S18*). However, it is unknown whether these genes are stably expressed in early developmental stages ranging from immature oocytes to expanded blastocyst stage embryos.

Most gene expression studies in preimplantation development have focused on murine models, but where mouse embryos form an egg cylinder, human and porcine embryos have a planar morphology [21], and where human and murine embryos show invasive implantation at the blastocyst stage, porcine embryos have a loose diffuse non-invasive placenta [22]. There are also some clear differences between preimplantation embryos at the molecular level. Human embryos differ in surface specific antigens expression compared to mouse [23], and in pigs, expression of the pluripotency marker *OCT4 *is, contrarily to human and mouse, not restricted to the inner cell mass [24]. These clear differences in mammalian early embryonic development could also have an effect on the validity of reference genes between species. Such species dependency of good reference genes has lately been demonstrated in differentiating mouse and human embryonic stem cells, cells that are closely related to preimplantation embryos [25]. To validate candidate reference genes in a non-mouse model, the potential of seven genes to be used as internal controls during early porcine embryonic development was investigated, using three separate pools of developmental stages ranging from germinal vesicle stage oocytes to blastocyst stage embryos.

## Results

### RT-PCR

**Figure 1:**
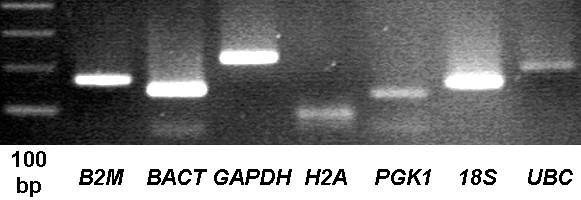
**PCR results of candidate reference genes**. PCR products were run on a 2% agarose gel. Amplicons were of the expected sizes (B2M 166 bp, BACT 141 bp, GAPDH 219 bp, H2A 83 bp, PGK1 126 bp, S18 149 bp, UBC 186 bp). 100 bp = 100 base pair ladder.

For each developmental stage, porcine oocytes and embryos with good morphology [26] were collected from three independent *in vitro *cultures [see Additional file 1], and RNA was isolated separately from these biological replicates. β-2-microglobulin (*B2M*), beta-actin (*BACT*), glyceraldehyde-3-phosphate dehydrogenase (*GAPDH*), histone 2α (*H2A*), phosphoglycerate kinase 1 (*PGK1*), 18S ribosomal RNA (*S18*), and ubiquitin (*UBC*) were chosen as candidate reference genes and used for RT-PCR on oocytes. These genes are regularly used to normalise mRNA transcript levels and at least five of the genes have previously been used as reference genes in early development (*BACT *[27], *GAPDH *[28], *H2A *[29], *S18 *and *UBC *[30]). Amplicons were of the expected sizes (Figure 1) and their specificity was confirmed by sequence analysis (data not shown). Optimal annealing temperature was determined using a temperature gradient ranging from 50 to 65°C (Table 1). For each biological replicate, three technical replicates were run in all qRT-PCR experiments, and all samples for one gene product were run on one 96-well plate to minimize inter-experimental variation. Dilution curves of all candidate reference genes showed an average amplification efficiency of 100.2% (min. 86%, max 113.3%) and an average coefficient of determination (R^2^) of 0.992. Single distinctive peaks in the melt curves confirmed specific amplification of the gene of interest. All reference genes were consistently detected in all samples except one metaphase-2 sample, which was excluded from further analysis. This reliable detection indicates integrity of the cDNA samples. Starting quantities were based on the gene specific standard curves and calculated with MylQ software. The -RT levels of *B2M, BACT, GAPDH*, and *PGK1*, were below detectable levels. *S18 *was detected at an average level of 0.05% in -RT samples, *UBC *was detected at an average of 0.007% and *H2A *was detected at an average of 1.27% compared to their RT counterparts. The -RT values were subtracted from their RT counterparts.

**Table 1:** Primer details used for qRT-PCR

Gene	Genbank Accession number	Sequence	Ta (°C)	amplicon size (bp)
*B2M*	NM_213978	5'-TTCACACCGCTCCAGTAG-3' 5'-CCAGATACATAGCAGTTCAGG-3'	59.5	166
*BACT*	AY550069	5'-CATCACCATCGGCAACGAGC-3' 5'-TAGAGGTCCTTGCGGATGTC-3'	55.8	141
GAPDH	AF017079	5'-TCGGAGTGAACGGATTTG-3' 5'-CCTGGAAGATGGTGATGG-3'	51.1	219
*H2A*	BX921568	5'-GCTGTTGGGCAAAGTCAC-3' 5'-GGCTCTCCGTCTTCTTGG-3'	54.3	83
*PGK1*	AY677198	5'-AGATAACGAACAACCAGAGG-3' 5'-TGTCAGGCATAGGGATACC-3'	56.4	126
*S18*	NR_002170	5'-GGCTACCACATCCAAGGAAG-3' 5'-TCCAATGGATCCTCGCGGAA-3'	58.7	149
*UBC*	M18159	5'-TTCGTGAAGACCTTGACTG-3' 5'-GGACTCCTTCTGGATGTTG-3'	51.1	186

Ta = optimal annealing temperature

### GeNorm analysis

Several statistical tools are available to identify stably expressed genes, but since studies have not found large differences between statistical tools such as geNorm, NormFinder, and Bestkeeper [25,31], only one of these applications was used here to calculate gene expression stability [19]. For each gene, Ct values of unknown samples were transformed into the log of the starting quantities with the formula obtained from the standard curve, thereby taking into account the efficiency of the PCR reaction. Raw starting quantities were analysed with geNorm to determine gene expression stability over the different developmental stages, which resulted in a gene expression stability measure *M *for each gene (Table 2). Stepwise exclusion of unstable genes and subsequent recalculation of the average *M*-values, results in a ranking of the genes based on their *M*-values, with the two most stable genes, with the lowest *M*-values, leading the ranking [see Additional file 2] [19]. This stepwise elimination of the least stable genes revealed that *GAPDH, PGK1, S18*, and *UBC *were the 4 most stable genes.

**Table 2:** Ranking of genes by expression stability; less stable genes have higher M-values.

Gene	*M*-value
1: GAPDH	1.278
2: *UBC*	1.300
3: *PGK1*	1.312
4: *S18*	1.352
5: *H2A*	1.650
6: *BACT*	1.912
7: *B2M*	2.540

**Figure 2:**
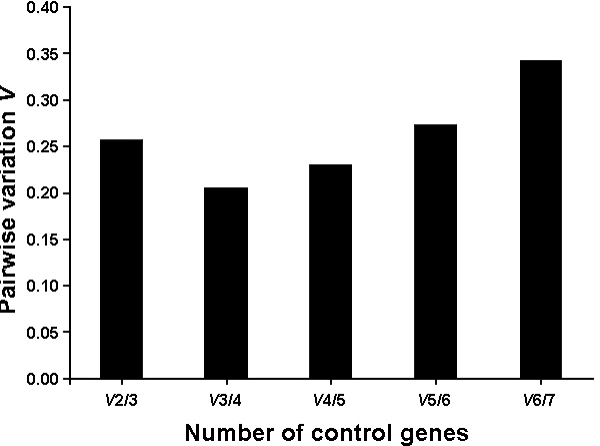
**Optimal number of reference genes**. Pair wise variation of normalization factors after successive inclusion of less stable genes determined the optimal number of reference genes. On the left-most side is the pair wise variation when the number of genes is enlarged from 2 to 3 (V2/3). Stepwise inclusion of less stable genes generates the next data points. Inclusion of a 4th gene has a significant effect on the normalization factors. Inclusion of a 5th, 6th, or 7th gene has a negative effect on the pair wise variation value and reflects the average expression stability M of these genes.

The geometric mean of the expression levels of the 2 best reference genes, *GAPDH *and *UBC*, was used to calculate normalisation factors. To reveal the optimum number of reference genes, it was determined whether stepwise inclusion of less stable genes significantly affected the normalisation factors. This pair wise variation (*V*) showed that inclusion of a fourth reference gene had a significant effect on the normalisation factors, but including a fifth gene did not improve the normalisation factors (Figure 2). Therefore, the least stable genes *B2M, BACT*, and *H2A *were not very suitable for normalisation. *GAPDH *and *PGK1 *play important roles in the glycolytic pathway and are potentially co regulated, but removal of either of these genes from the analysis did not affect the ranking of the genes by stability (Table 3). *In vitro *produced porcine embryos are vulnerable to polyspermy. In this study, the susceptibility to polyspermy was minimized by using sow oocytes instead of those from pre-pubertal gilts, [32,33] and by addition of porcine follicular fluid to the in vitro maturation medium [34]. Exclusion of the transcriptionally active blastocyst stages from the analysis resulted in merely minor influences on gene ranking (Table 3). Therefore, the same panel of reference genes can be used to normalize gene expression from the germinal vesicle stage oocyte to the 4-cell stage embryo, with *GAPDH, PGK1, UBC*, and *S18 *as the best four genes for normalisation, to be preferred over *H2A*, B2M, and *BACT*. Amplification of a large product might influence PCR efficiency and the final ranking of a gene. In this study, however, there was not a correlation between product size and *M*-value [see Additional file 3] or between product size and efficiency [see Additional file 4], indicating that the ranking of genes by stability was not a methodological artefact. Since the -RT levels for *H2A *were relatively high in some samples, the ranking of reference genes was also calculated when this gene was excluded, but exclusion of this gene left the ranking intact except for *UBC*, which was repositioned from first to fourth in rank (Table 3).

**Table 3:** Ranking of genes by expression stability.

All genes included	Minus *GAPDH*	Minus *PGK1*	Minus *H2A*	Minus Blastocysts
1: *GAPDH*	2: *UBC*	2: *UBC*	1: *GAPDH*	1: *GAPDH*
2: *UBC*	3:*PGK1*	**1: *GAPDH***	3:*PGK1*	**3: *PGK1***
3: *PGK1*	4:*S18*	4:*S18*	4:*S18*	**2: *UBC***
4: *S18*	5:*H2A*	5:*H2A*	**2: *UBC***	4:*S18*
5: *H2A*	6: *BACT*	6: *BACT*	6: *BACT*	5:*H2A*
6: *BACT*	7:*B2M*	7:*B2M*	7:*B2M*	**7: *B2M***
7: *B2M*	X	X	X	**6: *BACT***

In the first column, all genes and developmental stages were included in the analysis, and in the following columns the genes GAPDH, PGK1, or H2A were excluded, and in the last column, blastocyst stage embryos were excluded. The numbers represent ranking position of that gene when all genes are included. In bold are those genes from which the ranking is affected by the exclusion.

### Gene expression patterns

**Figure 3:**
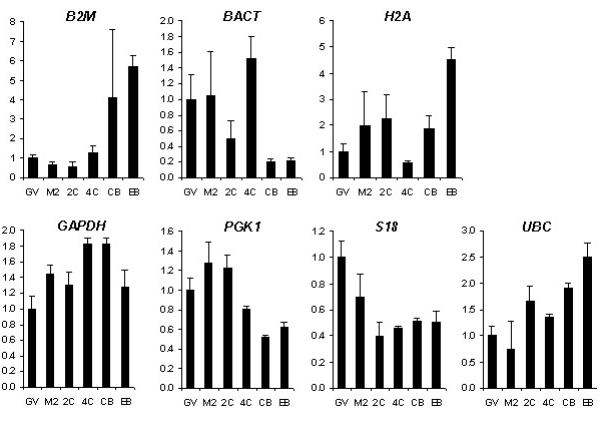
**Relative expression of BACT, H2A, B2M, GAPDH, PGK1, S18, and UBC at different stages of porcine embryonic development**. Top row: regulated genes; bottom row: stably expressed genes. X-axis: developmental stage (GV = germinal vesicle stage, M2 = metaphase-2 stage, 2C = 2-cell stage, 4C = 4-cell stage, CB = early cavitating blastocyst, EB = expanded blastocyst). Y-axis: normalized relative expression. Data was normalized to the geometric mean of 4 stably expressed genes (18S, PGK1, GAPDH, UBC) as determined by geNorm analysis. For each developmental stage, the normalized expression value was subsequently divided by the normalized expression value of the germinal vesicle stage. Error bars represent SEM.

In porcine early developmental stages *B2M, BACT*, and *H2A*, were not expressed at consistent levels. The expression pattern of these genes was studied by normalising the qRT-PCR data of these genes with the normalisation factors calculated by geNorm and based on the four most stable genes (Figure 3, top row). *B2M *expression levels in GV, M2, 2-cell, and 4-cell stage embryos were similar. Early blastocysts showed an increase in *B2M *expression, and highest expression levels were seen in expanded blastocysts. *BACT *showed stable expression in the oocyte stages, was downregulated at the 2-cell stage, showed up regulation at the 4-cell stage and was downregulated again in the blastocyst stages. *H2A *expression was upregulated in the M2 stage compared to the GV stage. The M2 and the 2-cell stage showed similar levels of *H2A *expression, and at the 4-cell stage, *H2A *expression was downregulated. Early blastocysts showed increased expression and expression was highest in expanded blastocyst stage embryos. Expression patterns of stably expressed genes are also presented (Figure 3, bottom row).

## Discussion

Gene expression studies often rely on an internal standard that shows stable expression in the cell types or tissues examined, independent of the differentiation or biological state of the cell. Identifying a good reference gene in preimplantation embryonic development is however hindered by significant fluctuations in the transcriptomes. Moreover, within mammals there is clear morphological and molecular biological variation between embryos of different species such as mouse, pig and human. In the present study, the usefulness of genes as reference genes in early porcine development was tested. *GAPDH *and *PGK1 *were identified as the genes with the most stable expression, followed by *S18 *and *UBC*, whereas *B2M, BACT*, and *H2A *demonstrated to be inferior reference genes in this non-mouse model. *In vitro *produced porcine embryos are susceptible to polyspermy. In this study, sow oocytes were used instead of those from pre-pubertal gilts, in order to minimize the generation of polyspermic embryos [32,33]. Moreover, exclusion of the transcriptionally active blastocyst stages from the analysis resulted in merely minor influences on gene ranking. There was not a correlation between product size and *M *value, or between product size and PCR efficiency, indicating that the ranking of genes by expression stability is not a methodological artefact, as described previously for amplicons below 200 base pairs [35].

Several studies have attempted to identify good reference genes in mammalian oocytes and preimplantation embryos [36-39]. Goossens et al. used geNorm to test reference genes in bovine preimplantation embryos, but unfortunately this study excludes oocytes and starts the developmental range from the 2-cell stage onwards [36]. Other studies have included oocytes in their ranges of developmental stages, and in those studies, the approaches were highly similar in that the numbers of oocytes/embryos were kept equal in RNA isolations. The RNA was subsequently used as a template for cDNA synthesis, and a fixed volume of cDNA was used in each PCR reaction. The best reference genes were considered those genes that showed least variation between developmental stages [37-39]. However, due to the nature of preimplantation development the quantity of RNA differs between stages. For example, 2-cell stage mouse embryos have less total RNA than germinal vesicle stage oocytes, since ~90% of maternal RNA will be degraded and the embryonic genome is mostly inactive. Genes that show constant expression throughout all stages are genes that somehow escape mRNA degradation from the GV stage to the 2-cell stage and are barely transcribed at later stages. Normalisation with such a gene will result in the absolute value that a gene is up- or downregulated. However, it is unclear whether this up- or downregulation is biologically significant, because the entire transcriptome might show a similar up- or downregulation. Therefore, in this study, appropriate reference genes were defined as those genes that represent the total RNA content, which is in line with methods such as Northern Blotting and RNAse protection assays. This difference in method partly explains the discrepancy in findings between previous studies and the current one. For example, *H2A *has previously been advocated as a good reference gene in bovine and murine preimplantation development, because of its unaffected mRNA levels during preimplantation development [37,38]. In contrast, in this study, the average expression stability *M *of *H2A *was high and this gene was therefore excluded from the set of reference genes on which the normalisation factors were based. Moreover, *GAPDH, PGK1, S18*, and *UBC *have previously been rejected as good reference genes for the reason that their gene products show high fluctuations in absolute copy numbers in early development [37-39]. In this study, however, these genes are advocated as reference genes, because their fluctuations reflect the fluctuations in the transcriptomes. With this method, it should be kept in mind that apparent up- or downregulation of a certain gene might be caused by changes in total RNA abundance and not reflect increased or decreased transcription.

The four best candidate reference genes *GAPDH, PGK1, S18*, and *UBC *were stably expressed in spontaneously differentiating human embryonic stem cells as well. Moreover, these genes showed stable expression in differentiating mouse embryonic stem cells, with the exception *of S18 *[25]. Although thorough testing of reference genes in any new experimental set-up is advocated, it is not unlikely that these genes can also be used to normalise gene expression data of preimplantation embryos and embryonic stem cells, enabling a comparison between these two closely related systems. In mouse peri-implantation embryos ranging from 3.5 days post coitum (dpc) blastocysts to 9.5 dpc neurulating embryos *GAPDH, PGK1*, and *S18 *show unstable expression [25], which is an indication that these genes should not be used to study post-implantation development.

By pair wise variation the optimal number of reference genes was determined at four. Therefore, normalisation factors were derived from the best four reference genes and these factors were subsequently used to calculate the gene expression patterns of regulated genes. *B2M *plays a role in expression of MHC class I antigen on cells. *B2M *showed increased expression in blastocyst stages, which is consistent with the role that *B2M *has in the proper expression of blastocyst Major Histocompatibility Complex, to protect fetal trophoblast cells from maternal NK cells [40]. Expression of *BACT*, a major component of microfilaments of the cytoskeleton of eukaryotic cells, was downregulated at 2-cell stage embryos compared to the oocyte stages, indicating that *BACT *mRNA is actively degraded or translated between these stages. *BACT *mRNA peaked at the 4-cell stage, which coincides with the porcine embryonic genome activation and might reflect the cytoskeletal changes when embryos switch from cleavage divisions to cell proliferation in later stages. *H2A *is one of the 5 main histone proteins, which are involved in the structure of chromatin. Chromatin structure has an important role in epigenetic gene regulation and is probably involved in coordinating gene activity in early embryonic stages [41]. *H2A *expression increased in expanded blastocyst stages, which suggests changes in chromatin structure and possibly nuclear reprogramming at that stage of development. In summary, mRNA abundance of *B2M, BACT*, and *H2A *showed distinct temporal regulation from oocyte maturation to early embryo development.

## Conclusion

Analysis of transcription profiles of 7 candidate reference genes in early porcine embryonic development revealed that *GAPDH, PGK1, S18*, and *UBC *showed stable expression and can therefore be used as reference genes. Since *GAPDH *and *PGK1 *both have roles in glycolysis, it is advised not to use both genes for normalisation, unless independent stability of these genes is made clear. Therefore, a selection of either *GAPDH or PGK1 *together with *S18 *and *UBC *is proposed. To our knowledge, this is the first evaluation of reference genes that reflect total RNA content in early mammalian embryonic development from oocyte to blastocyst.

## Methods

### Oocyte maturation, IVF, embryo culture

All incubations described below took place in a humidified atmosphere of 38.5°C and 5% CO_2_. Recovery, *in vitro *maturation and fertilization of porcine oocytes and subsequent *in vitro *culture of porcine embryos proceeded as previously described [42]. In short, sow ovaries were collected from a regional slaughterhouse and cumulus oocyte complexes (COCs) were aspirated from antral follicles of 2–6 mm. Oocytes of equal size and three or more layers of compact cumulus were selected and transferred to maturation medium, containing NCSU-23 medium [43], supplemented with 10% porcine follicular fluid, 0.57 mM cysteine, 25 mM β-mercaptoethanol, 10 IU/ml eCG, (Chorulon, Intervet, Boxmeer, the Netherlands) and 10 lU/ml hCG (Folligonan, Intervet). After 24 hr culture, the COCs were transferred to maturation medium excluding eCG and hCG and cultured for an additional 18 hrs. After culture, COCs were denuded and transferred to Tris-buffered IVF-medium, containing 113.1 mM NaCl, 3 mM KC1, 20 mM Tris, 11 mM D-glucose, 1 mM caffeine, 5 mM sodium pyruvate, 7.5 mM CaCl_2_, 0.1% (w/v) bovine serum albumine, (BSA; Sigma-Aldrich Chemie, Zwijndrecht, the Netherlands) and 1% pen/strep. IVF was performed with fresh semen from two randomly selected boars at a concentration of 1000 cells/oocyte. Next day, the presumptive zygotes were cultured in IVC medium, which contained NCSU-23 and 0.4% BSA.

### RNA extraction and reverse transcription

Total RNA was isolated from denuded germinal vesicle stage oocytes, denuded metaphase 2 stage oocytes (as confirmed by the presence of one polar body), 2-cell stage embryos, 4-cell stage embryos, early blastocysts and expanded blastocysts, using the RNeasy minikit (Qiagen, Venlo, the Netherlands). RNA yield is lower in oocytes and cleavage stage embryos compared to blastocyst stage embryos and therefore, 40 oocytes/embryos per sample were collected for the early stages and 10 blastocysts were pooled for every sample. First-strand cDNA was synthesised with Superscript II (Invitrogen, Groningen, the Netherlands) and random primers were used to prime reverse transcription of RNA. As negative controls, mixtures were prepared without reverse transcriptase. Reaction mixtures with samples were incubated for 1 hr at 42°C and subsequently for 5 min at 80°C, chilled on ice and stored at -20°C.

### Quantitative RT-PCR

Preceding qRT-PCR amplification, primers were designed using Primer Select software (DNAstar, Madison, WI, USA) and Beacon Designer 4 (PREMIER Biosoft International, Palo Alto, CA, USA) (Table 1). Primers were tested on cDNA of *in vitro *produced embryos. The amplicons were run on a 2% agarose gel and products were sequenced to test the specificity of the primers. Subsequent PCR was performed in a Bio-Rad iCycler (Bio-Rad, Veenendaal, the Netherlands). Gene transcripts were quantified using iQ SYBR Green supermix (Bio-Rad). The optimal annealing temperature of the primers was tested experimentally with a temperature gradient. A separate reaction was run for each gene and a standard curve of tenfold dilution ranging from 10 pg to l ag supplemented each run. All points of the standard curve and all samples were run in triplets as technical replicates. In each run 1 μl of cDNA was used as template for amplification per reaction. The sample was added to 24 μl of reaction mixture, containing 12.5 μl H_2_O, 11.25 μl iQ SYBR Green supermix (Bio-Rad), and 0.5 mM of both forward and reverse primers (Isogen, Maarssen, the Netherlands). The thermal cycling profile started with a 3-min dwell temperature of 94°C, followed by 40 cycles of 30 sec at 94°C, 30 sec at the primer specific annealing temperature, 30 sec at 72°C, and a final step at which fluorescence was acquired. After 40 cycles, the program continued with a post-dwell of 1 min at 94°C. Finally, a melt curve was generated by temperature increments of 0.1°C starting from 65 to 100°C, with fluorescence acquisition after each step. Data was analysed with My-IQ software (Bio-Rad), which for all samples calculated the starting quantities of all candidate reference genes, based on the standard curves for these genes.

## Authors' contributions

EWK was responsible for the experimental procedures and was the primary author for the manuscript. LP contributed to the IVF experiments. HTAT provided support throughout the experimental process. HPH and BC supervised the study design. BAJR supervised study design, analyses and writing. All authors read and approved the final manuscript.

## Supplementary Material

Additional file 1 **Figure S1: Representative pictures of porcine oocytes and embryos collected for qRT-PCR**. (A) Germinal vesicle stage, (B) metaphase-2 stage, (C) 2-cell stage, (D) 4-cell stage, (E) early (cavitating) blastocyst, (F) expanded blastocyst. Size bars: a-f 50 μm. Click here for file

Additional file 2 **Figure S2: Average expression stability M after stepwise exclusion of the least stable gene**. On the left-most side is the average expression stability M for all genes, with the least stable gene within that group on the x-axis. Exclusion of this gene from the analysis generates the next data point. After stepwise exclusion of the least stable genes, the two best genes, which cannot be further ranked, remain and are depicted on the rightmost side of the graph. Click here for file

Additional file 3 **Figure S3: Product size and M-value are not correlated**. Product size is plotted against the stability factor M. M-values of the genes are not correlated to the product size of the PCR reactions (R2 = 0.0196). Click here for file

Additional file 4 **Figure S4: Product size and amplification efficiency are not correlated**. Product size is plotted against efficiency of the PCR reaction as an the average of the plus RT and the minus RT run. Average efficiencies are not correlated to the product size (R2 = 0.0199). Click here for file
