# Supplementary figures and images for: Validation of reference genes for quantitative RT-PCR studies in porcine oocytes and preimplantation embryos
Source: BMC Dev Biol. 2007 May 31;7:58. doi: 10.1186/1471-213X-7-58 (PMC1896162; doi:10.1186/1471-213X-7-58)

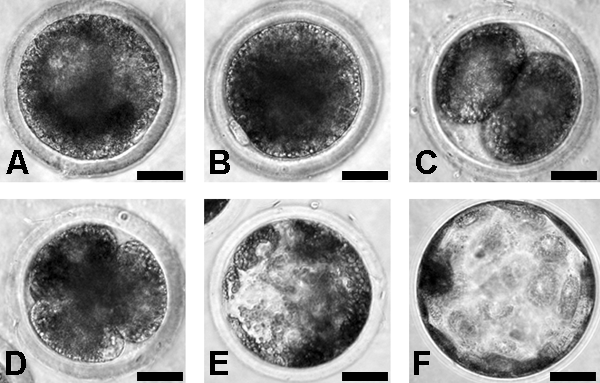

Supplement: Additional file 1 — Figure S1: Representative pictures of porcine oocytes and embryos collected for qRT-PCR. (A) Germinal vesicle stage, (B) metaphase-2 stage, (C) 2-cell stage, (D) 4-cell stage, (E) early (cavitating) blastocyst, (F) expanded blastocyst. Size bars: a-f 50 μm. [file 1471-213X-7-58-S1.tiff]

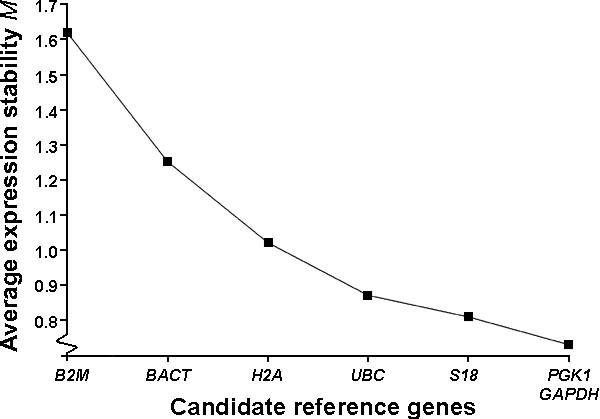

Supplement: Additional file 2 — Figure S2: Average expression stability M after stepwise exclusion of the least stable gene. On the left-most side is the average expression stability M for all genes, with the least stable gene within that group on the x-axis. Exclusion of this gene from the analysis generates the next data point. After stepwise exclusion of the least stable genes, the two best genes, which cannot be further ranked, remain and are depicted on the rightmost side of the graph. [file 1471-213X-7-58-S2.tiff]

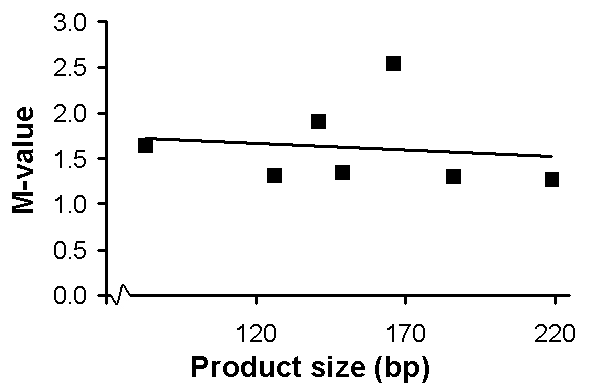

Supplement: Additional file 3 — Figure S3: Product size and M-value are not correlated. Product size is plotted against the stability factor M. M-values of the genes are not correlated to the product size of the PCR reactions (R2 = 0.0196). [file 1471-213X-7-58-S3.tiff]

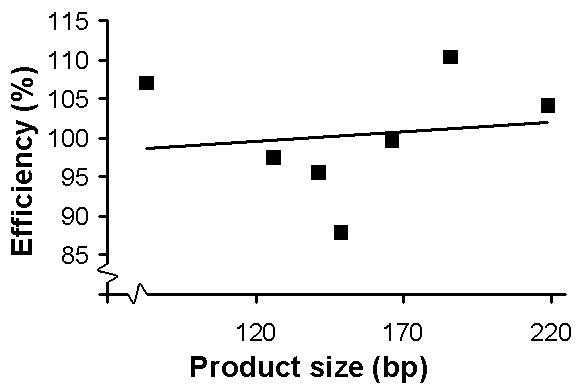

Supplement: Additional file 4 — Figure S4: Product size and amplification efficiency are not correlated. Product size is plotted against efficiency of the PCR reaction as an the average of the plus RT and the minus RT run. Average efficiencies are not correlated to the product size (R2 = 0.0199). [file 1471-213X-7-58-S4.tiff]
